# Characterization of Potent SMAC Mimetics that Sensitize Cancer Cells to TNF Family-Induced Apoptosis

**DOI:** 10.1371/journal.pone.0161952

**Published:** 2016-09-12

**Authors:** Kate Welsh, Snezana Milutinovic, Robert J. Ardecky, Marcos Gonzalez-Lopez, Santhi Reddy Ganji, Peter Teriete, Darren Finlay, Stefan Riedl, Shu-ichi Matsuzawa, Clemencia Pinilla, Richard Houghten, Kristiina Vuori, John C. Reed, Nicholas D. P. Cosford

**Affiliations:** 1 Sanford Burnham Prebys Medical Discovery Institute, 10901 N. Torrey Pines Rd, La Jolla, CA, 92037, United States of America; 2 Torrey Pines Institute for Molecular Studies, 3550 General Atomics Ct, San Diego, CA, 92121, United States of America & 11350 SW Village Parkway, Port St. Lucie, FL, 34987, United States of America; The University of Texas MD Anderson Cancer Center, UNITED STATES

## Abstract

Members of the Inhibitor of APoptosis (IAP) protein family suppress apoptosis within tumor cells, particularly in the context of immune cell-mediated killing by the tumor necrosis factor (TNF) superfamily cytokines. Most IAPs are opposed endogenously by the second mitochondrial activator of caspases (SMAC), which binds to selected baculovirus IAP repeat (BIR) domains of IAPs to displace interacting proteins. The development of SMAC mimetics as novel anticancer drugs has gained impetus, with several agents now in human clinical trials. To further understand the cellular mechanisms of SMAC mimetics, we focused on IAP family members cIAP1 and cIAP2, which are recruited to TNF receptor complexes where they support cell survival through NF-κB activation while suppressing apoptosis by preventing caspase activation. We established fluorescence polarization (FP) assays for the BIR2 and BIR3 domains of human cIAP1 and cIAP2 using fluorochrome-conjugated SMAC peptides as ligands. A library of SMAC mimetics was profiled using the FP assays to provide a unique structure activity relationship (SAR) analysis compared to previous assessments of binding to XIAP. Potent compounds displayed mean inhibitory binding constants (K_i_) of 9 to 27 nM against the BIR3 domains of cIAP1 and cIAP2, respectively. Selected compounds were then characterized using cytotoxicity assays in which a cytokine-resistant human tumor cell line was sensitized to either TNF or lymphotoxin-α (LT-α). Cytotoxicity correlated closely with cIAP1 and cIAP2 BIR3 binding activity with the most potent compounds able to reduce cell viability by 50%. Further testing demonstrated that active compounds also inhibit RIP1 binding to BIR3 of cIAP1 and cIAP2 *in vitro* and reduce steady-state cIAP1 protein levels in cells. Altogether, these data inform the SAR for our SMAC mimetics with respect to cIAP1 and cIAP2, suggesting that these IAP family members play an important role in tumor cell resistance to cytotoxicity mediated by TNF and LT-α.

## Introduction

Defects in the regulation of apoptosis underlie many disease processes, including cancer [1]. In most malignancies, insufficient apoptosis contributes to pathological cell accumulation whilst also promoting resistance to chemotherapy and various therapeutic interventions. Caspases, a family of intracellular cysteine proteases, are the effectors of apoptosis [2]. These proteases are present as inactive zymogens in essentially all mammalian cells. Some caspases are inhibited by members of the inhibitor of apoptosis proteins (IAP) family [3]. IAPs contain a structural motif called the baculovirus IAP repeat (BIR) domain that participates in the binding of active caspases. Most IAPs also operate as E3 ligases due to the presence of a RING domain, which interacts with ubiquitin conjugating enzymes (UBCs). Certain IAPs also bind via their BIR domains to other classes of protein targets, including proteins involved in signal transduction pathways leading to activation of NF-κB and the stress kinases of the MAPK pathway [4, 5].

Several IAPs are suppressed by endogenous proteins, such as the second mitochondrial activator of caspases (SMAC) [6]. A minimum required tetrapeptide sequence (AVPI) from SMAC (AVPI) binds a groove on the BIR domain of IAPs, thus dislodging caspases [7]. The ability of the AVPI tetrapeptide to neutralize IAPs and enable apoptosis has sparked multiple drug discovery efforts aimed at producing peptidyl and non-peptidyl small molecules with drug-like properties as candidate therapeutics for cancer (reviewed in [8]).

One of the challenges with the SMAC mimetic strategy is defining the repertoire of BIR domains that bind these compounds and elucidating the cellular consequences thereof. In this regard, the XIAP protein has served as the prototype for the design of all SMAC mimetics thus far. The XIAP protein consists of three tandem BIRs, followed by an ubiquitin-binding domain (UBA) and a RING domain which functions as an E3-ligase [9, 10]. BIR2 of XIAP binds caspases-3 and -7, while BIR3 binds caspase-9 [11]. SMAC tetrapeptides interact with both BIR2 and BIR3 of XIAP, typically with approximately 10-fold higher binding affinity for BIR3 compared with BIR2 [12]. XIAP plays an especially important role in suppressing apoptosis induced by tumor necrosis factor (TNF) family cytokines including Fas Ligand (CD95L) and TNF-related apoptosis-inducing ligand (TRAIL) [13, 14].

The IAP family members cIAP1 and cIAP2 have an architecture similar to XIAP, with 3 tandem BIR domains, a UBA domain, a RING domain and a caspase activation and recruitment domain (CARD) [15]. As with XIAP, the BIR2 and BIR3 domains of cIAP1 and cIAP2 also bind caspases and SMAC [6, 16, 17]. In contrast to XIAP, the dominant role of cIAP1 and cIAP2 in apoptosis regulation appears to occur in the context of TNF signaling via TNFR1 (CD120a), where these proteins play an essential role in NF-κB induction and suppression of TNF-induced apoptosis [18]. In this regard, the BIR3 domains of cIAP1 and cIAP2 bind the TNFR1 complex kinase (RIP1) and catalyze non-canonical ubiquitination of RIP1 to promote signaling events leading to NF-κB induction and suppression of caspase activation [19, 20].

Recently, we described the design and synthesis of SMAC mimetic compounds and reported their interactions with the BIR2 and BIR3 domains of XIAP [21, 22]. A selection of these compounds were tested to determine effects on certain cancer cell lines, specifically their ability to sensitize tumor cells to the TNF-related apoptosis-inducing ligand (TRAIL) induced killing [23]. Here, we have extended the evaluation of these compounds to the SMAC-binding BIR2 and BIR3 domains of cIAP1 and cIAP2. Our findings reveal that the most potent compounds have higher affinity interactions with the BIR domains of cIAP1 and cIAP2 than with XIAP. Moreover, exemplary compounds sensitize tumor cell lines to the cytotoxic activity of the cytokines TNF and lymphotoxin-α (LT-α), which utilize the TNFR1 complex to transduce signals into cells. Additionally, active compounds inhibit RIP1 binding to the BIR3 domains of cIAP1 and cIAP2. Our findings therefore suggest that it is possible to tailor SMAC mimetics for inhibition of IAP family members cIAP1 and cIAP2, thereby modulating tumor responses to TNF and LT-α.

## Materials and Methods

### Recombinant proteins

Proteins used in this study are either the GST-tagged versions of cIAP1 (aa144-260) and cIAP2 BIR2 (aa129-246) purified from glutathione-Sepharose resin or the His6-tagged versions of cIAP1 (aa257-356) and cIAP2 BIR3 (aa243-333) purified using Ni-NTA resin. Bacterially expressed recombinant proteins were purified to >70% homogeneity for GST-fusions and >95% for His6-fusions, as estimated by Coomassie blue dye staining of proteins analyzed by SDS-polyacrylamide gel electrophoresis (SDS-PAGE). GST-tagged proteins were dialyzed into 50 mM Tris [pH8.0] containing 1 mM DTT at concentrations of ~0.5 mM and stored frozen at -80°C in aliquots. His6-tagged proteins were recovered in 50 mM Tris [pH 8.0], 100 mM NaCl, 10% glycerol, 300 mM imidazole at ~0.5 mM and also stored at -80°C in aliquots. Protein concentrations were determined using the BioRad Protein Assay reagent.

### Peptides

The heptapeptide AVPIAQK was synthesized by previously established methods [24]. In brief, the rhodamine labeled SMAC peptide (TPI 1237–22) was synthesized using the simultaneous multiple peptide synthesis approach on methylbenzhydrylamine polystyrene resin with Boc chemistry. The incorporation of the rhodamine was accomplished by coupling lissamine rhodamine B sulfonyl chloride (L20, Molecular Probes) to the side chain of the C-terminal lysine of the heptapeptide. The rhodamine labeled peptide was purified by reverse phase high performance liquid chromatography (HPLC). The purity and identity were characterized by liquid chromatography and mass spectrometry. The sulforhodamine used for the synthesis contains *ortho* and *para*-isomeric monosulfonyl chlorides, which results in the sulfonamides that are both *ortho* and *para* to the xanthylium ring system. The purified product was identified as the *para*-isomer based on the stability of its color at elevated pH [25].

Fluorescence intensity of SMAC-rhodamine was determined in 25 mM Hepes [pH 7.5] containing 1 mM tris(2-carboxyethyl)phosphine hydrochloride (TCEP) and 0.005% Tween 20, showing linearity in the concentration range from 0.12 to 125 nM. Based on these findings we chose to work with SMAC-rhodamine at 20 nM in subsequent experiments. Data were collected on a Molecular Devices 96:384 Analyst HT plate reader in fluorescence intensity mode with excitation at 530 nm and emission at 580 nm using a dichroic mirror at 565 nm.

### Fluorescence polarization (FP) assays

Solutions for assessing SMAC-rhodamine binding to various BIR domains were empirically optimized. The FP assays were conducted in black 384-well plates (Greiner Bio-One #784076) using various buffered solutions. For cIAP1-BIR3, the assay buffer was 25 mM Hepes [pH 7.5] containing 40 mM β-glycerol phosphate, 1 mM TCEP, and 0.005% Tween 20, typically with 20 nM SMAC-rhodamine. For cIAP1-BIR2, cIAP2-BIR2 and cIAP2-BIR3, assays were performed in 25 mM Hepes [pH 7.5] containing 1 mM TCEP and 0.005% Tween 20, typically with 20 nM SMAC-rhodamine. Plates were incubated for 5 minutes at room temperature and data were collected using an Analyst HT plate reader (Molecular Devices 96:384) in fluorescence polarization mode with excitation filter at 530 nm, emission filter at 580 nm, and the dichroic mirror at 565 nm. EC_50_ values for SMAC-rhodamine binding to BIR domains were determined with a nonlinear fit using data analysis software (GraphPad Prism, GraphPad Software, Inc.), and estimated K_d_ values were calculated as described [26].

For IC_50_ determinations, FP assays were empirically optimized for each BIR, achieving signal-noise ratios > 10, an assay window > 9-fold, and Z’ factors > 0.5. For cIAP1-BIR2, assays were conducted using 25 mM Hepes [pH 7.5] containing 1 mM TCEP, 0.005% Tween 20, ~1 μM cIAP1-BIR2, and 20 nM SMAC-rhodamine. For cIAP1-BIR3, assays were performed using the same buffered solution, except 40 mM β-glycerol phosphate was added and the cIAP1-BIR3 concentration was reduced to 50 nM. For cIAP2-BIR2, assays included a buffer containing 25 mM Hepes [pH 7.5] containing 1 mM TCEP, 0.005% Tween 20, 1 μM cIAP2-BIR2, and 20 nM SMAC-rhodamine. These assay conditions were also used for cIAP2-BIR3 with the exception that the target protein concentration was 125 nM. Data were collected as described above. IC_50_ values were determined with a nonlinear fit using Prism software. K_i_ values were determined from the IC_50_ values using an online program developed by Nikolovska-Coleska *et al*. [26].

### Cytotoxicity assays

Cellular cytotoxicity experiments were conducted in white 96-well flat bottom plates (Greiner Bio-One #655074). PC-3 prostate cancer cells were seeded into microtiter wells in 90 μL RPMI medium with 10% fetal calf serum and antibiotics at 5,000 cells per well, and then incubated overnight at 37°C with 5% CO_2_/95% air. The next day, compounds diluted into the same culture medium and added in 10 μL aliquots to achieve various final concentrations, maintaining a uniform total volume per well. Either TNF (at 10 nM final) or LT-α (at ~90 pM final) resuspended in 10 μL of RPMI with 10% fetal calf serum and antibiotics was added to wells or an equal volume of medium was applied for the control well. The plates were then further incubated for 24 h. Subsequently, cell viability was assessed by the addition of 25 μL per well of Cell-Titer-Glo reagent (Promega, Inc). Plates were read using a Luminoskan Ascent luminometer. IC_50_ values (representing the concentration of the SMAC mimetic analogue that reduced cell viability by 50% compared to cytokine alone) were determined by nonlinear fit using Prism software (GraphPad, La Jolla, CA).

### Immunoblotting

To assess the impact of compounds on cIAP1 protein levels in cells, 40,000 MDA-MB-231 cells were cultured in complete medium (RPMI with 10% fetal calf serum and antibiotics) overnight at 37°C in 5% CO_2_/95% air. The following day compounds at 5 μM (or an equivalent volume of diluent control) were added. After 6 h, cells were recovered by centrifugation, lysed in SDS-sample buffer, and equivolume aliquots of lysates were analyzed by SDS-PAGE/Immunoblotting using antibodies specific for human/mouse cIAP (pan) Mab (clone 315301; R&D Systems #MAB3400) and β-actin (Sigma-Aldrich #A5441) by an enhanced chemiluminescence (ECL) method, essentially as described [27, 28].

### RIP1 binding

To assess the impact of compounds on cIAP1 and cIAP2 binding to RIP1, 700,000 HEK293T cells were seeded into 100 mm cell-culture plates and transfected the next day with 6 μg of plasmid DNA, myc-RIP1 in pRK5 vector. After 24 h, cells were lysed in NP-40 buffer (20 mM Tris-HCl [pH 7.5], 135 mM NaCl, 1 mM EDTA, 0.5% NP-40, 10% glycerol) supplemented with EDTA-free protease inhibitors (Roche) and 1 mM DTT. Lysates from 6 plates were pooled and divided into equal 300 μL aliquots, into which 7 μg of purified GST-fusion proteins (GST-cIAP1-BIR3 or GST-cIAP2-BIR3) were added in the presence of various concentrations of SMAC mimetic compounds. The tubes were rocked overnight at 4°C, and the next day 20 μL of glutathione-Sepharose beads (GE Healthcare) was added to each tube and the tubes were rocked for an additional 2 h at 4°C. The GST-fusion proteins were recovered by centrifugation at 2,300 g for 30 sec, then washed 3-times in NP-40 lysis buffer. Finally, 20 μL of 2X Laemmli buffer was added and the samples were boiled, then fractionated by SDS-PAGE, followed by immunoblotting using anti-myc antibody (Roche) for detection of myc-RIP1 and anti-GST antibody (BD Biosciences, La Jolla, CA) for detection of GST-fusion proteins.

## Results

### Establishing FP assays for measuring SMAC peptide binding to BIRs of cIAP1 and cIAP2

**Fig 1:**
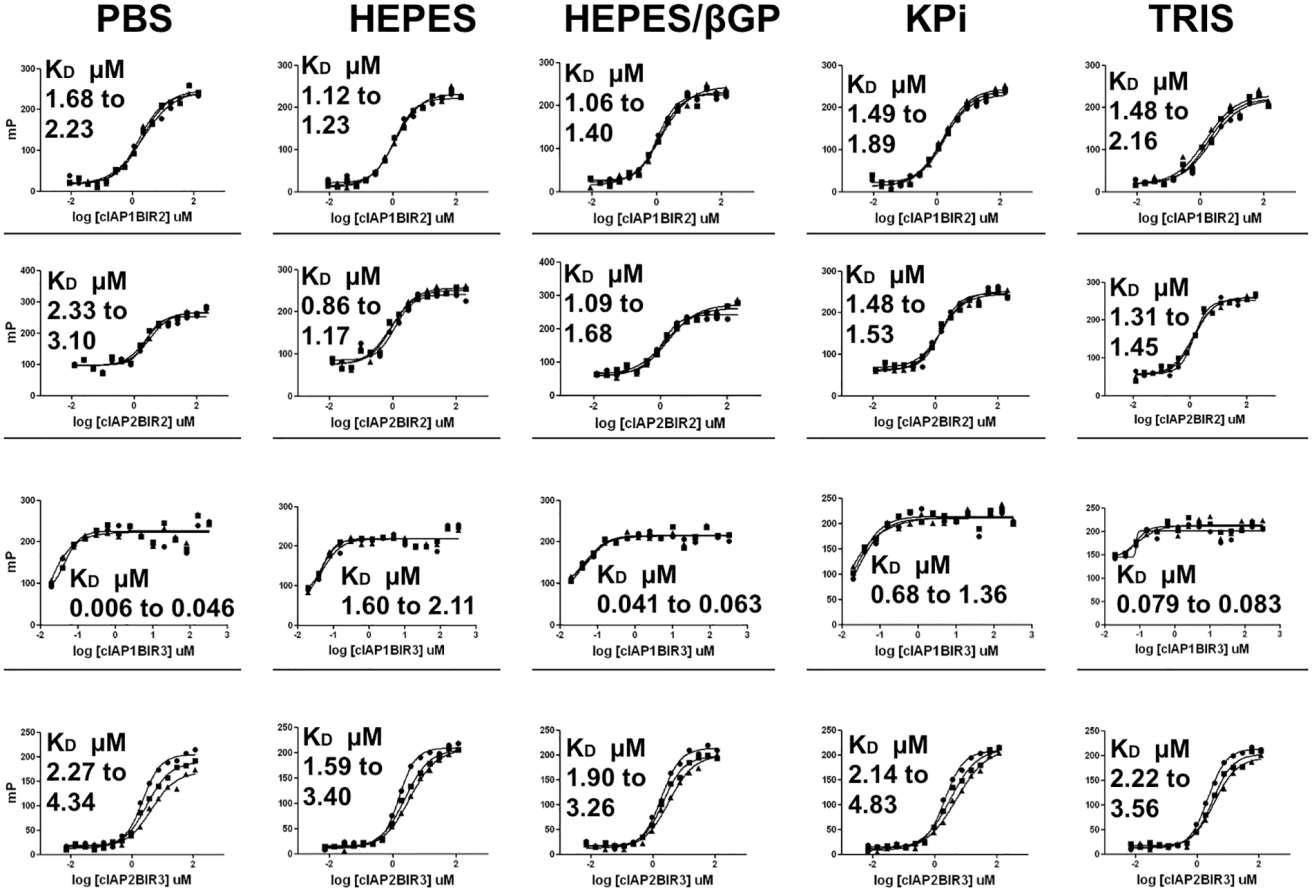
Effect of buffer on K_D_ of SMAC peptide binding to BIR domains. All assays contained TCEP at 1 mM, 0.005% Tween 20 and SMAC-rhodamine at 20 nM. The buffers used were PBS @ pH 7.4, 25 mM HEPES @ pH 7.5, 25 mM HEPES @ pH 7.5 with 20 mM β-glycerol phosphate, 10 mM Potassium Phosphate @ pH 7.4, or 50 mM TRIS @ pH 7.5. Proteins were diluted into 25 mM HEPES @ pH 7.5 with 1 mM TCEP. FPA data were collected on the Analyst at 0, 30 and 60 min. Time overlays are plotted in the figure. K_D_s were determined in Prism.

We established FP assays for assessing the interaction of SMAC mimetic compounds with the BIR domains of cIAP1 and cIAP2. For this purpose, the SMAC-binding BIR2 and BIR3 domains of human cIAP1 and cIAP2 were expressed in bacteria and purified. The SMAC heptapeptide AVPIAQK conjugated at the C-terminal lysine with rhodamine was employed as the ligand for the FP assays. An examination of the dependence of fluorescence intensity on the concentration of SMAC-rhodamine proved to be linear at concentrations up to 125 nM, and we set 20 nM as the peptide concentration for displacement by SMAC mimetic compounds, which provided a good signal window while firmly remaining in the linear range of signal detection. The concentration dependence of binding of the various BIRs from cIAP1 and cIAP2 to SMAC peptide was measured by FP assays in the presence of various buffered solutions in an effort to optimize assay performance (Fig 1). The pH was held in the physiological range of 7.4 to 7.5, comparing assay performance for 5 different buffers: PBS, Hepes, Hepes/β-glycerol phosphate, potassium phosphate, or Tris. Assay stability was assessed by collecting data at 30 min and 60 min and the K_D_ values were determined for each BIR domain (cIAP1-BIR2 [~ 1.3 μM], cIAP1-BIR3 [~ 0.14 μM], cIAP2-BIR2 [~ 1.31 μM], and cIAP2-BIR3 [~ 0.38 μM]) at each time point. Other substituents were empirically included, which consisted of 1 mM TCEP to maintain a reducing environment (due to the chelation of zinc within BIR domains by cysteine residues) and 0.005% Tween 20 (to reduce risk of target protein aggregation induced by promiscuous compounds). Though most assay solutions tested yielded satisfactory results, we selected the Hepes-buffer solution for further experiments, consisting of 25 mM Hepes [pH 7.5], 1 mM TCEP, 0.005% Tween 20, and 20 nM SMAC-rhodamine for cIAP1-BIR2, cIAP2-BIR2 and cIAP2-BIR3. For cIAP1-BIR3, the same solution was employed, with addition of 40 mM β-glycerol phosphate.

**Fig 2:**
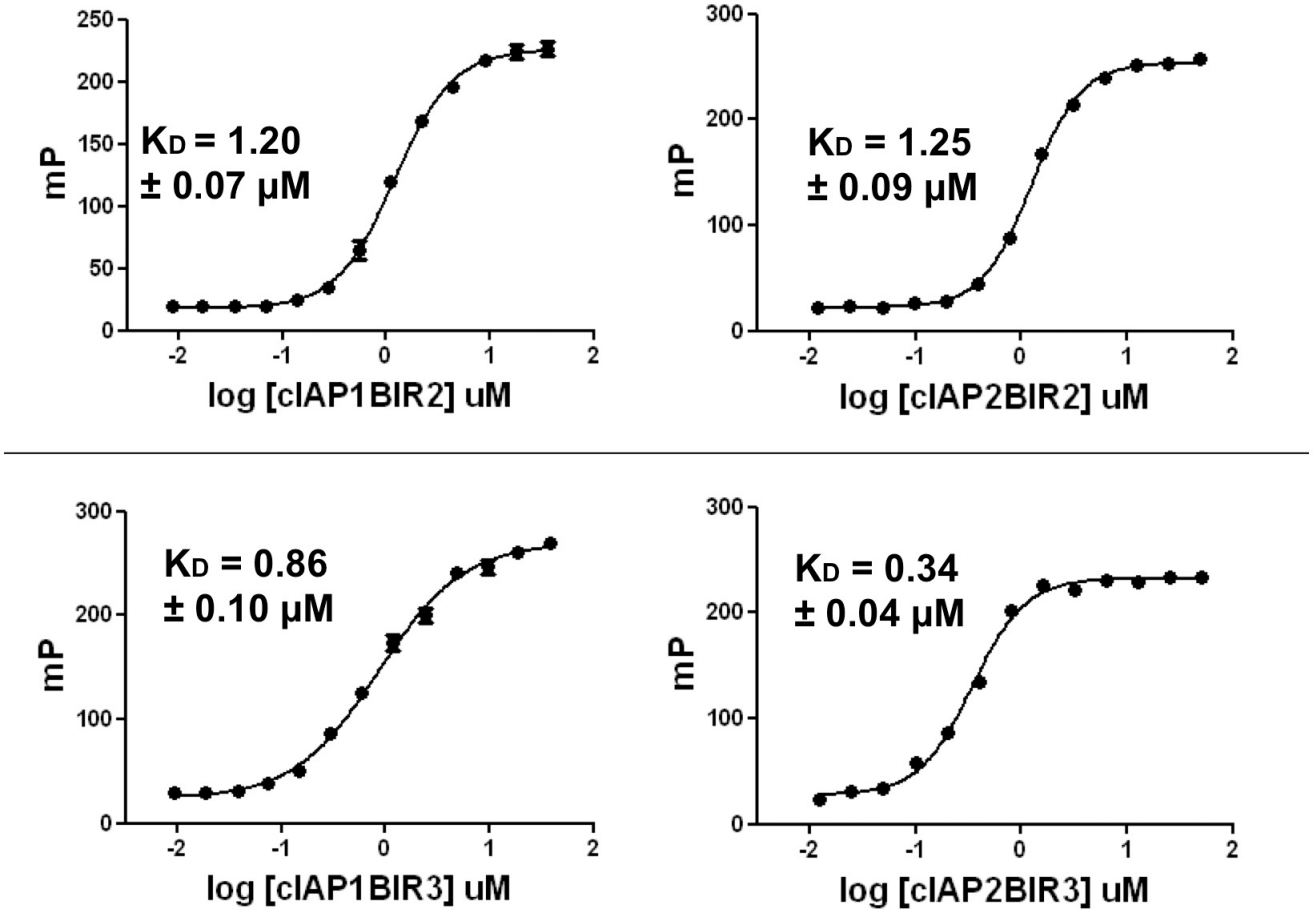
K_D_ determination of SMAC-rhodamine binding to BIR2 and BIR3 domains of cIAP1 and cIAP2. Data were for assay conditions consisting of 25 mM Hepes @ 7.5, 1 mM TCEP, 20 nM SMAC-rhodamine with varying concentrations of various BIR domains. For cIAP1-BIR3, assays included 40 mM β-glycerol phosphate. Plates were read on the Analyst and observed mP were plotted against the log of protein concentration.

The EC_50_ values under these optimized conditions were determined, and K_D_ values were estimated from the data (24). The BIR2 domains of cIAP1 and cIAP2 had very similar estimated K_D_ values (apparent K_D_) of 1.20 ± 0.07 μM and 1.25 ± 0.09 μM (mean ± standard deviation), respectively. In contrast, SMAC peptide bound slightly tighter to the BIR3 domains, with the estimated K_D_ values of the BIR3 domains of 0.86 ± 0.10 μM for cIAP1-BIR3 and 0.34 ± 0.04 μM for cIAP2-BIR3 (Fig 2). Thus, the affinity of SMAC peptide for BIR2 versus BIR3 domains of cIAP1 and cIAP2 is only marginally different, in contrast to XIAP where it has been observed that BIR3 binds SMAC peptide ~10 fold more tightly than BIR2 [12].

**Fig 3:**
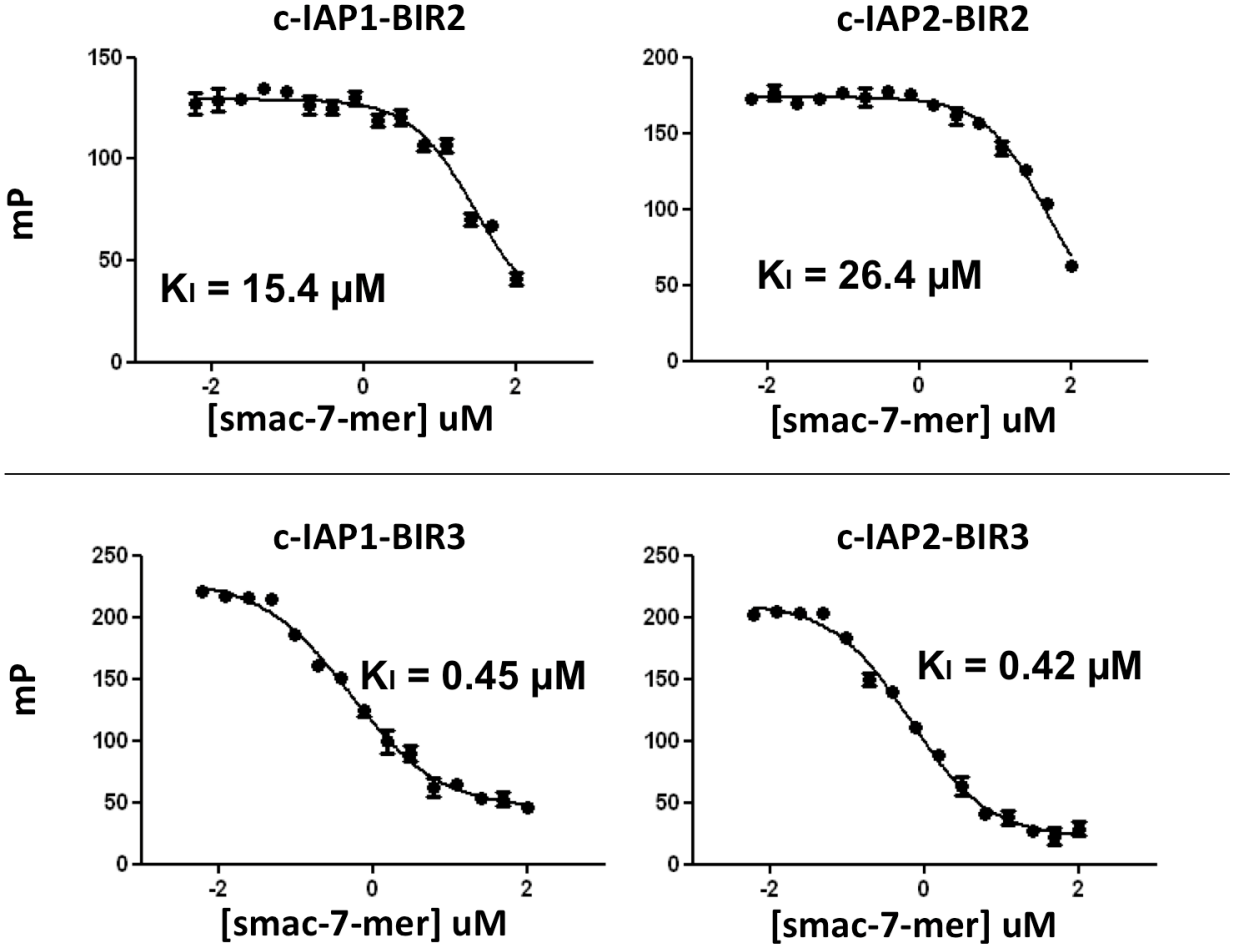
Competition of SMAC-7-mer with SMAC-rhodamine. Assays conditions were 25 mM Hepes @ pH 7.5, 1 mM TCEP, 0.005% Tween 20 and 20 nM SMAC-rhodamine. Where cIAP1-BIR3 was present, 40 mM β-glycerol phosphate was also present in the assay. Proteins were present at ~50 nM for cIAP1-BIR3, 125 nM for cIAP2-BIR3, and at 1 μM for both cIAP1-BIR2 and cIAP2-BIR2. SMAC peptide (AVPIAQK) ranged between ~6 nM and 100 μM.

Next, we determined the K_i_ values for competitive displacement of SMAC-rhodamine ligand from BIR domains by unconjugated (non-fluorescent) SMAC peptide. The protein concentrations of BIR domains were empirically adjusted to achieve signal:noise ratios >10, assay window of >9-fold, and Z’ factors >0.5. Based on previously determined K_D_ values, we settled on ~50 nM for cIAP1-BIR3, 125 nM for cIAP2-BIR3, and 1 μM for cIAP1-BIR2 and cIAP2-BIR2. Unlabeled SMAC heptapeptide (AVPIAQK) was added between 6.1 nM and 100 μM for competition analysis. IC_50_ values were determined from the curves shown in Fig 3 and K_i_ values were calculated [26].

### Evaluation of activity of SMAC mimetic compounds against BIR2 and BIR3 domains of cIAP1 and cIAP2

**Fig 4:**
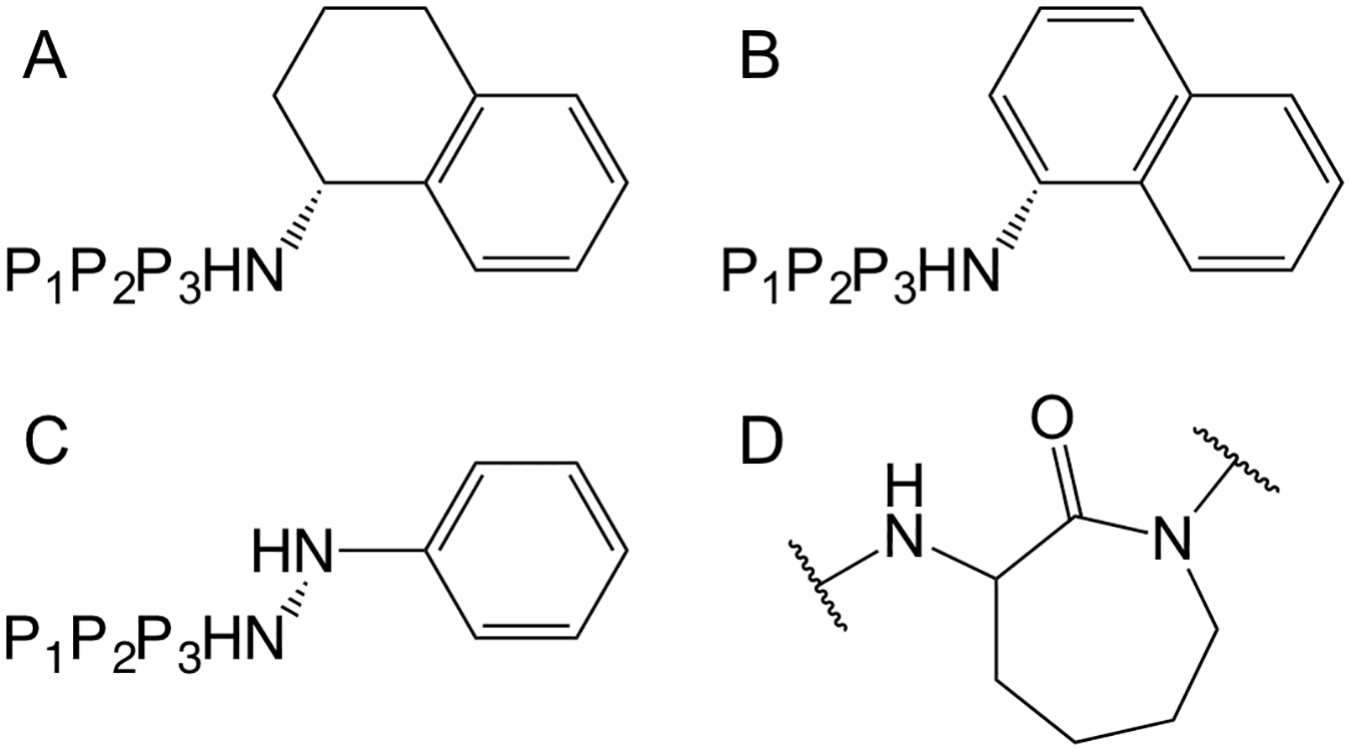
Condensed structures (A,B, and C) of the compounds series described in Tables 1, 2 and 3. (D) Structure of P_2_ substituent of compound **18**.

We examined the effects on SMAC-rhodamine peptide binding to cIAP1 and cIAP2 BIR2 and BIR3 domains using a library of synthesized compounds that we have previously described in detail, and characterized with respect to XIAP binding [21–23]. These SMAC mimetics represent tripeptide derivatives that we evolved using an iterative medicinal chemistry strategy from the prototype AVPI tetrapeptide. The SAR data from the present study are reported in Tables 1–3 showing the impact on binding affinities of substituting the various residues in P_1_-P_4_. The SMAC mimetic analogues are organized into subgroups with either tetrahydronaphthyl (Table 1 and Fig 4A), naphthyl (Table 2 and Fig 4B), or phenylhydrazine (Table 3 and Fig 4C) C-terminal capping groups that replace the P4 terminal isoleucine residue of AVPI. With the C-terminal capping groups in place, modifications of the P_2_ (valine) and P_3_ (proline) residues of the resulting tripeptides were synthesized and their activity compared. Because structural studies have shown the critical importance of L-alanine for the P_1_ position [12, 29], and the ability of N-methyl-alanine to effectively substitute [30], we fixed P_1_ as either L-alanine or N-methyl-L-alanine for most of the SMAC mimetics. In some cases other moieties at P_1_ were included to purposely generate less active and inactive analogues as controls for subsequent bioactivity studies (see below).

**Table 1:** Binding affinities of tetrahydronaphthyl series SMAC mimetic compounds for BIR domains of cIAP1 and cIAP2^a^.

	*P*_*1*_	*P*_*2*_	*P*_*3*_	*cIAP1 BIR3 K*_*i*_ *μM*	*cIAP1 BIR2 K*_*i*_ *μM*	*cIAP2 BIR3 K*_*i*_ *μM*	*cIAP2 BIR2 K*_*i*_ *μM*
**1**	N-Me-Ala	Val	Pro	0.02	0.84	0.04	2.34
**2**	N-Me-Ala	Ile	Pro	0.04	1.11	0.09	2.08
**3**	N-Me-Ala	AABA	Pro	0.06	2.34	0.06	5.27
**4**	N-Me-Ala	Val	Ala	0.11	2.21	0.12	4.55
**5**	N-Me-Ala	Leu	Pro	0.06	1.33	0.12	4.35
**6**	N-Me-Ala	Asp-Ome	Pro	0.08	2.16	0.19	6.38
**7**	N-Me-Ala	Asp	Pro	9.4	> 57.2	12.6	> 56.7
**8**	N-Me-Ala	Gln	Pro	0.08	1.26	0.15	4.07
**9**	N-Me-Ala	Val	Nω-nitro-arg	0.05	0.81	0.07	2.62
**10**	N-Me-Ala	Val	Cbz-Orn	0.28	1.41	0.22	2.07
**11**	N-Me-Ala	Val	Gln	0.72	3.25	0.55	5.50
**12**	N-Me-Ala	Val	Cbz-Lys	0.20	3.31	0.50	5.46
**13**	N-Me-Ala	Val	Orn	0.09	1.47	0.07	3.75
**14**	N-Me-Ala	Val	Ser	0.23	3.09	0.30	6.81

Assays were run in 25 mM Hepes @ pH 7.5/1 mM TCEP/0.005% Tween 20/20 nM SMAC-rhodamine with protein ranging from 50 nM to 1 μM. In the case of cIAP1-BIR3, 40 mM β-glycerol phosphate was also present. IC_50_ values were observed and the corresponding K_i_s were calculated. P_1_, P_2_ and P_3_ groups are the stated L-amino acids or AABA (alpha-aminobutyric acid), Cbz-Orn [(benzylcarboxy)carbamate]-L-ornithine, Cbz-Lys [(benzylcarboxy)carbamate]-L-lysine.

^a:^ see Fig 4A for a condensed structure of this series.

**Table 2:** Binding affinities of naphthyl series SMAC mimetic compounds for BIR domains of cIAP1 and cIAP2^a^.

*ID*	*P*_*1*_	*P*_*2*_	*P*_*3*_	*cIAP1 BIR3K*_*i*_ *μM*	*cIAP1 BIR2K*_*i*_ *μM*	*cIAP2 BIR3K*_*i*_ *μM*	*cIAP2 BIR2K*_*i*_ *μM*
**15**	N-Me-Ala	Val	Ala	3.23	8.96	5.10	17.5
**16**	N-Me-Ala	Val	Pro	0.18	1.73	0.20	3.35
**17**	Ala	Val	Ala	1.23	3.77	0.98	5.98
**18**	N-Me-Ala	^b^	Ala	5.04	26.2	9.94	> 56.7
**19**	Ser	Val	Ala	4.37	8.33	17.2	22.4
**20**	AABA	Val	Ala	4.77	24.9	5.29	43.8
**21**	Ala	Val	N'-Cbz-Orn	0.85	2.77	0.99	4.79

Assays conditions contained 25 mM Hepes @ pH 7.5/1 mM TCEP/0.005% Tween 20/20 nM SMAC-rhodamine with protein concentrations ranging from 50 nM to 1 μM. In the case of cIAP1-BIR3, 40 mM β-glycerol phosphate was also present. IC_50_ values were observed and the corresponding K_i_s were calculated. P_1_, P_2_ and P_3_ are the stated L-amino acids, AABA (alpha-aminobutyric acid), or Cbz-Orn [(benzylcarboxy)carbamate]-L-ornithine.

^a:^ see Fig 4B for a condensed structure of this series.

^b:^ see Fig 4D for a structure of the substituent.

**Table 3:** Binding affinities of hydrazine series SMAC mimetic compounds for BIR domains of cIAP1 and cIAP2^a^.

*ID*	*P*_*1*_	*P*_*2*_	*P*_*3*_	*cIAP1 BIR3K*_*i*_ *μM*	*cIAP1 BIR2K*_*i*_ *μM*	*cIAP2 BIR3K*_*i*_ *μM*	*cIAP2 BIR2K*_*i*_ *μM*
**22**	N-Me-Ala	Val	Pro	0.26	3.62	0.56	5.43
**23**	N-Me-Ala	Val	Ala	6.13	7.39	8.29	13.6
**24**	N-Me-Ala	Val	Val	2.48	3.73	3.52	7.19
**25**	Ala	Val	Ala	3.20	4.45	3.07	7.44
**26**	N-Me-Ala	Val	Nω-nitro-arg	1.16	1.41	1.19	4.07
**27**	N-Me-Ala	Val	Val	2.65	3.09	4.8	10.1
**28**	N-Me-Ala	Val	Arg	1.97	4.39	1.42	15.4
**29**	N-Me-Ala	Glu	Ala	10.9	7.67	23.0	25.1
**30**	N-Me-Ala	Val	Ser	3.19	6.10	7.07	14.8
**31**	N-Me-Ala	Val	Cbz-Orn	0.98	1.70	1.29	2.93
**32**	N-Me-Ala	Val	Orn	4.01	3.82	2.17	10.9
**33**	N-Me-Ala	Val	Ser	5.54	4.37	7.96	11.5
**34**	Ala	Val	Ala	4.50	10.1	5.54	18.4
**35**	N-Me-Ala	Val	Lys	2.83	2.18	1.35	5.09
**36**	Ala	Val	Pro	0.34	6.11	0.57	12.9

Assays conditions contained 25 mM Hepes @ pH 7.5/1 mM TCEP/0.005% Tween 20/20 nM SMAC-rhodamine with protein concentrations ranging from 50 nM to 1 μM. In the case of cIAP1-BIR3, 40 mM β-glycerol phosphate was also present. IC_50_ values were observed and the corresponding K_i_s were calculated. P_1_, P_2_ and P_3_ are the stated L-amino acids or Cbz-Orn [(benzylcarboxy)carbamate]-L-ornithine.

^a:^ see Fig 4C for a condensed structure of this series.

For these studies, the SMAC mimetics were titrated into binding reactions using fixed concentration of SMAC-rhodamine and BIR domains, yielding IC_50_ values, from which K_i_ values were calculated [26]. Uniformly, the affinity of the SMAC mimetics was higher for the BIR3 domains of cIAP1 and cIAP2 than for the BIR2 domains (Tables 1–3), which is a characteristic observed previously for the XIAP BIR2 and BIR3 domains [21–23]. This reflects the higher affinity of the BIR3 domains for the wild-type SMAC peptide compared to BIR2. The capping group leading to the highest affinity cIAP1 and cIAP2 binders was the tetrahydronaphthyl. K_i_ values as low as 24 ± 1 nM were obtained for cIAP1-BIR3 and 40 ± 10 nM for cIAP2-BIR3 (*e*.*g*. compound **1**), when the P_1_-P_3_ positions consisted of the SMAC prototype N-methyl-alanine-valine-proline (Table 1). Other substitutions of P_1_, P_2_, or P_3_ reduced affinity for cIAP1 and cIAP2 BIRs, but several modifications resulted in only modestly impaired binding affinity. These substitutions included leucine, isoleucine, AABA (alpha-aminobutyric acid), Asp-OMe, and glutamine at P_2_ as well as N**ω**-nitro-Arg at P_3_. Thus, several substitutions at P_2_ are well tolerated, consistent with structural data that show the P_2_ residue side-chain projecting into the solvent [29, 31]. Moreover, our findings show that even the proline at P_3_ can be substituted with an unnatural amino acid (*e*.*g*. N**ω**-nitro-Arg), suggesting a path forward for generating more drug-like, less peptidyl compounds that target cIAP1 and cIAP2.

Similar trends were also observed for the naphthyl (Table 2) and hydrazine (Table 3) capping group series, though these molecules were an order of magnitude weaker in their ability to compete with wild-type SMAC peptide for binding to BIR domains of cIAP1 and cIAP2. The most potent compounds of these series consisted of the prototypical tripeptide N-methyl-alanine-valine-proline, with naphthyl (**16**) and hydrazine (**22**) substitutions at the C-terminus, which showed K_i_ values roughly 10-fold higher (weaker binding) than the same tripeptide capped with tetrahydronaphthyl. Moreover, substitutions at P_2_ or P_3_ residues in the naphthyl and hydrazine series were not as well tolerated compared to the tetrahydronaphthyl series.

### SMAC mimetics sensitize tumor cells to cytotoxicity of TNF and LT-α

The cellular activity of cIAP1 and cIAP2 inhibitors was evaluated through experiments where cytotoxicity of a human prostate tumor cell line (PC-3) was assessed in the presence or absence of cytokines TNF and LT-α. The experimental approach we used was to provide TNF or LT-α at a fixed concentration, which by itself was non-cytotoxic, then titrate SMAC mimetics into the cell cultures. As anticipated from our prior studies, none of the SMAC mimetic compounds showed appreciable cytotoxic activity by themselves on the cell lines tested. The compound series described in this work has been tested against a broad range of cell lines (data not shown and [23]) and single agent toxicity was only observed for SKOV3 ovarian tumor cells [32]. This agrees with the findings of Varfolomeev, Vince and Lalaoui, respectively [33–35].

**Fig 5:**
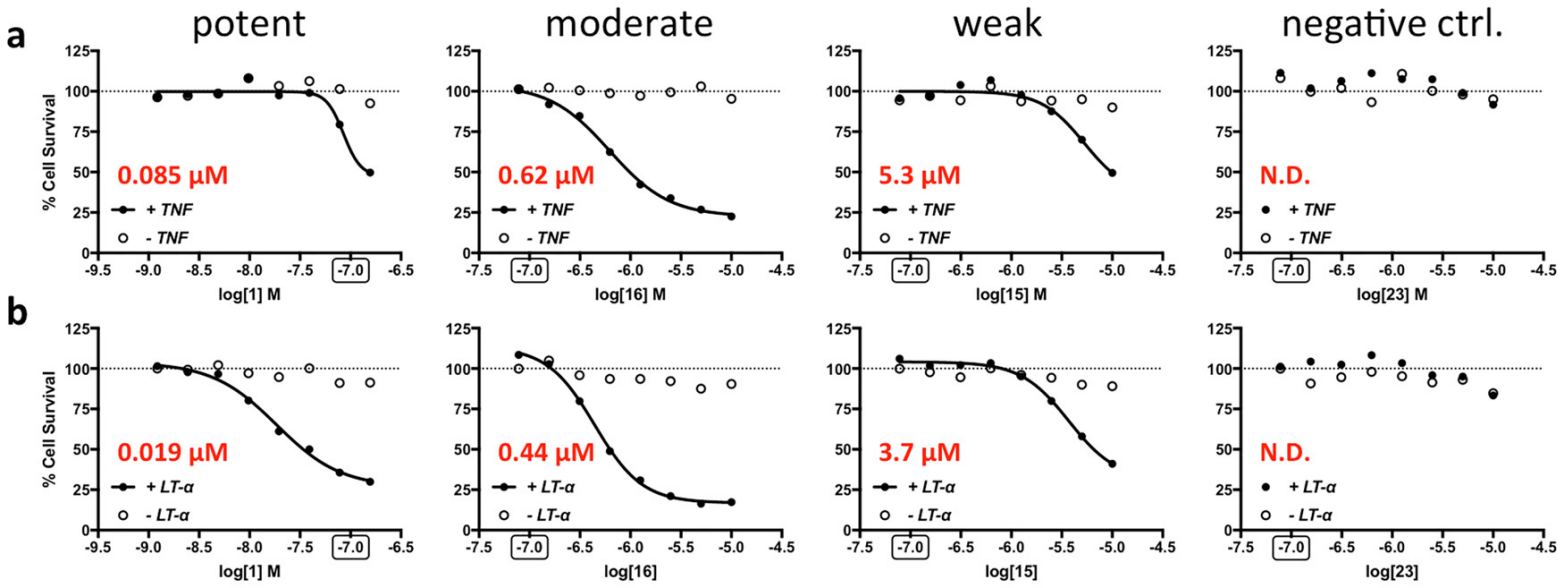
SMAC analogues sensitize PC-3 tumor cells to TNF and LT-α. Representative curves are shown in this figure for potent, moderate, weak and inactive analogues. Five thousand cells were seeded per well of 96 well plates and incubated overnight in culture medium. The following day, compounds were added and then 10 nM TNF or 90 pM LT-α was administered to the experimental wells, while media was added to control wells. Incubation was continued for 24 h. and then CellTiter-Glo was added to each well and the plates were read in a luminometer. EC_50_ values were determined with a nonlinear fit using Prism software. The most potent compounds were tested at a lower concentration range (see 100 nM point highlighted by box).

**Fig 6:**
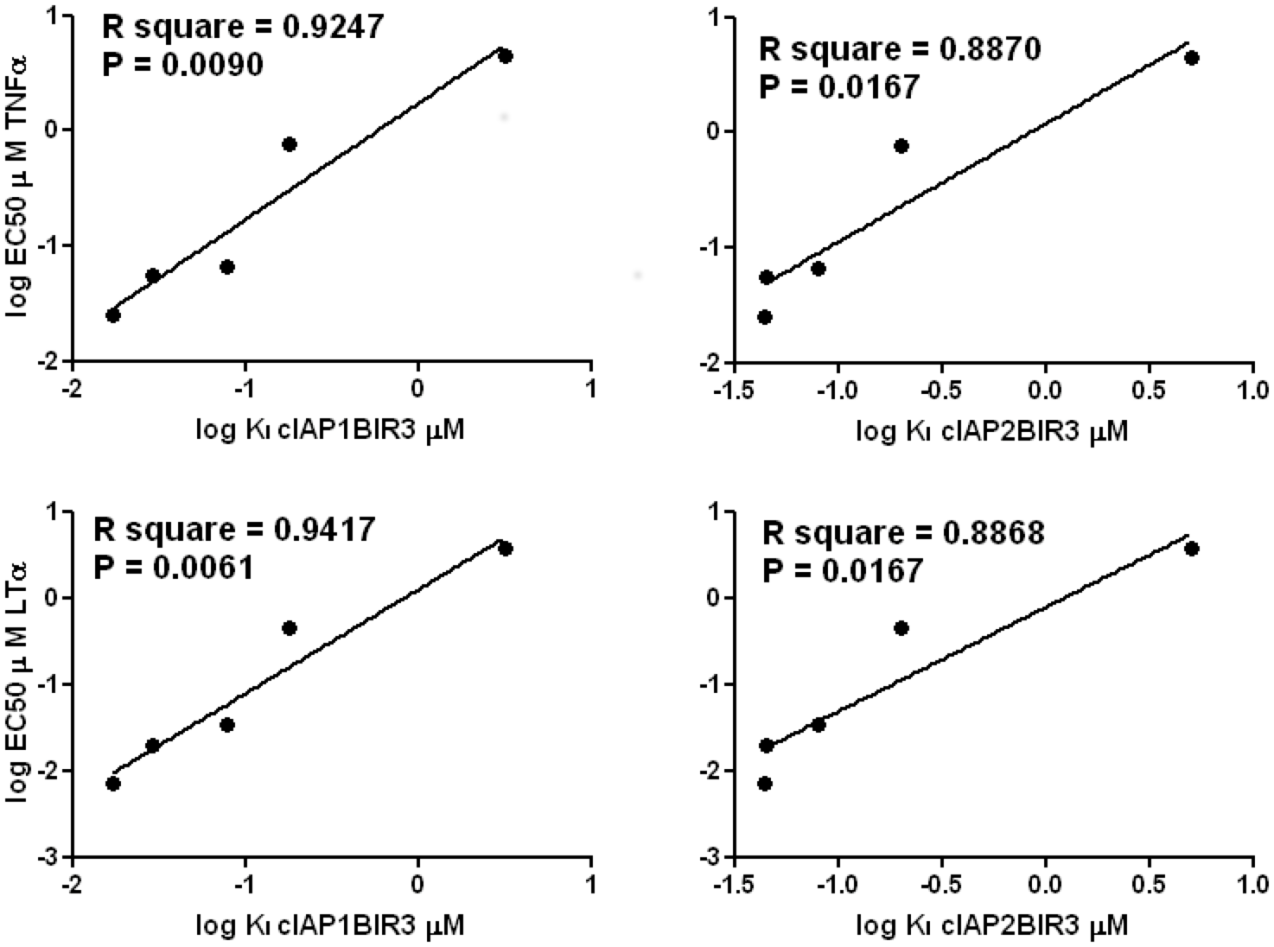
Correlation between K_i_ of the cIAP BIR domains and their EC_50_. Log of the K_i_ values for SMAC peptide displacement as measured by FPA was plotted against cell viability EC_50_ values using the data shown in Fig 5 for either TNF or LT-α. Correlations coefficient (r) and p-values are indicated.

In general, the ability of these compounds to bind cIAP1 and cIAP2 in FP assays correlated well with their ability to sensitize tumor cells to TNF or LT-α induced cell death. We compared compounds each with no activity (negative controls), weak activity (K_i_ = 1 to 10 μM), moderate activity (K_i_ = 100 nM to 1 μM), and potent activity (K_i_ = < 100 nM) in the FP assays and determined their ability to sensitize to cell death. For example, the potent compounds **1**, **37**, and **38** have K_i_ values against cIAP1-BIR3 ranging from 7 to 22 nM (Tables 1 and 4), while their cellular activity IC_50_ values for cytotoxicity against PC-3 cells ranged from 45 to 85 nM for TNF (Fig 5a) and 6.5 to 20 nM for LT-α (Fig 5b). In contrast, the weak compound **23** with a K_i_ value of 6.1 μM against cIAP1-BIR3 based on FPA showed essentially no cytotoxicity against PC-3 cells (Fig 5, Table 4). By comparison, the moderately potent compound, **15** (K_i_ value against cIAP1-BIR3 = 3.2 μM), showed modest cytotoxicity against PC-3 tumor cells with EC_50_ values of 5.3 and 3.7 μM for TNF and LT-α, respectively (Fig 5) [all data shown in Fig 5 can be found in S1 Prism]. Log-regression analysis showed a strong correlation between competitive binding to the BIR3 domains of the cIAPs and cytotoxic sensitizing activity (Fig 6).

**Table 4:** EC_50_ Determinations of selected and reference compounds with LT-α and TNF.

*ID*^a^	LT-α *EC*_*50*_ *μM*	TNF *EC*_*50*_ *μM*	*cIAP1 BIR3 K*_*i*_ *μM*	Ref.
**15**	3.69	4.46	3.2	**8q** [22]
**16**	0.44	0.8	0.2	**2b** [22]
**23**	IN^b^	IN^b^	6.1	**8h** [22]
**37**	0.01	0.03	0.02	[29]
**38**	0.02	0.06	0.03	[40]
**39**	0.03	0.07	0.08	**9q** [21]
**40**	IN^b^	IN^b^	> 92.4	^c^

^a:^ see Fig 9 for structures of the compounds in this table.

^b:^ EC_50_ too weak to be determined.

^c:^ inactive reference compound provided by M. Davis.

### Effects of SMAC mimetics on cIAP1 protein levels in cells

**Fig 7:**
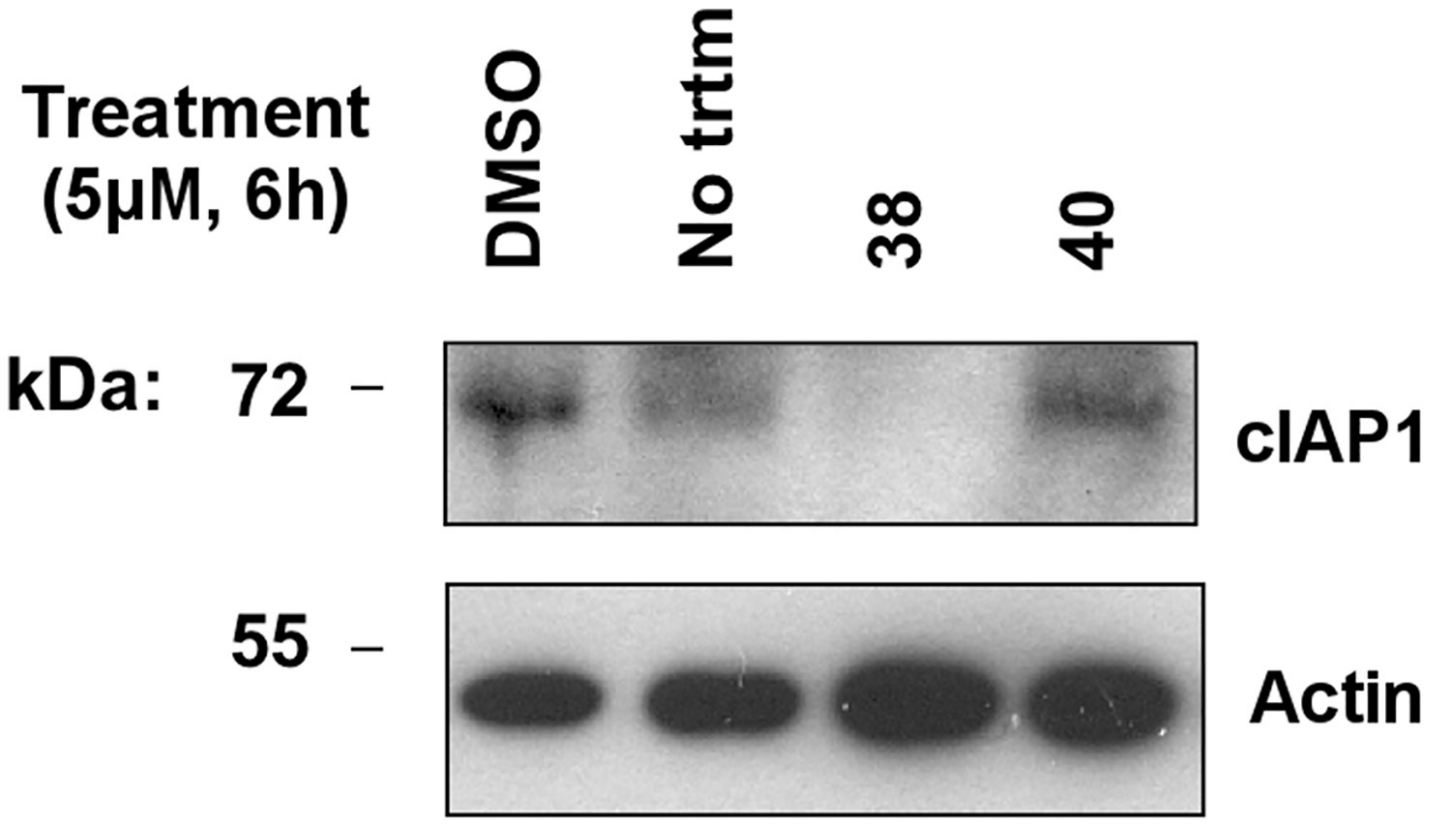
SMAC mimetic 38 induces degradation of cIAP1. MDA-MB-231 cells were seeded at 40,000 per well of 12 well plates and cultured overnight. The next day, cultures were either left untreated (No trtm) or were treated for 6 h. with DMSO, 5 μM of SMAC mimetic **38** or 5 μM of inactive analogue compound **40**. Cells were lysed in SDS-sample buffer and lysates were analyzed by SDS-PAGE/immunoblotting using antibodies specific for cIAP1 and beta-actin. Molecular weight markers are indicated in kilo-Daltons (kDa).

Various SMAC mimetics have been shown to stimulate self-directed E3 ligases of cIAP1 and cIAP2, resulting in their auto-ubiquitination and subsequent proteasomal degradation [33, 34]. We therefore evaluated the effects of cIAP1 and cIAP2 inhibitory SMAC analogues on levels of cIAP1 protein in MDA-MB-231 cells. Like most solid tumor cell lines, MDA-MB-231 cells contain abundant levels of cIAP1 protein but little cIAP2 protein. For these experiments, cells were cultured for 6 h in the presence or absence of SMAC mimetics (diluent control provided instead), comparing highly active versus inactive analogues. Fig 7 shows an example of the results for active compound **38** versus inactive analogue **40**. We observed that active compound **38** caused reductions of cIAP1 protein levels in cells, while inactive compound **40** did not, consistent with prior reports that SMAC mimetics stimulate the E3 ligase activity of cIAP1 and cIAP2 to induce their ubiquitination and subsequent degradation [36, 37].

### Active SMAC analogues inhibit RIP1 binding to cIAP1 and cIAP2

**Fig 8:**
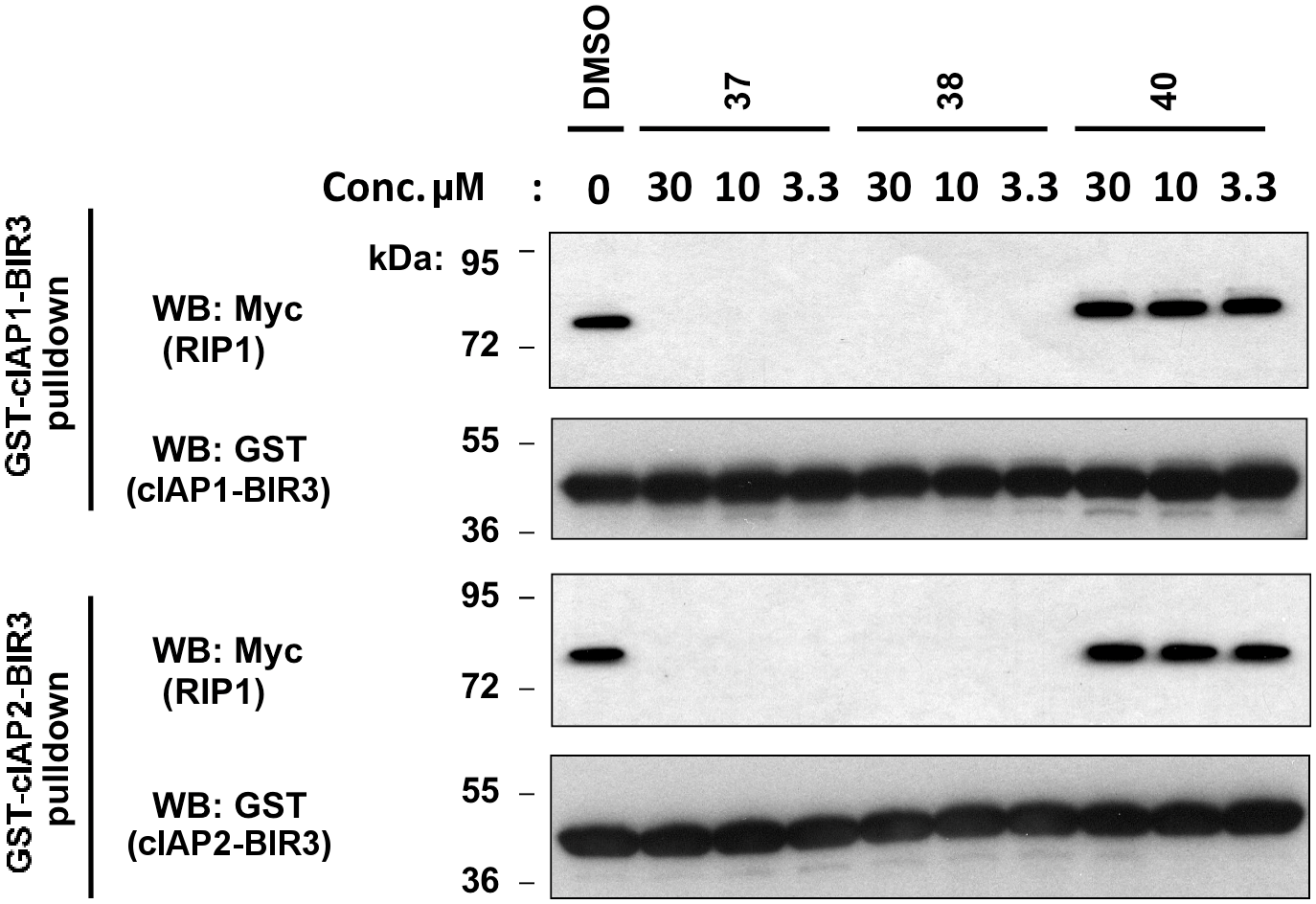
SMAC mimetics inhibit cIAP1 and cIAP2 binding to RIP1. HEK293T cells were transfected with myc-RIP1 plasmid and 24h later, cell lysates were prepared and divided into equal aliquots to which 7 μg of either GST-cIAP1-BIR3 or GST-cIAP2-BIR3 was added. Then, aliquots of either DMSO control or various concentrations of SMAC mimetics **37**, **38** or inactive analogue **40** were added. After overnight incubation, GST-cIAP1-BIR3 or GST-cIAP2-BIR3 proteins were recovered using glutathione-sepharose beads and the bound myc-RIP1 protein was detected by SDS-PAGE/immunoblotting using anti-myc antibody for detection of myc-RIP1 and anti-GST antibody for detection of GST-fusion proteins.

Recently, it was reported that cIAP1 and cIAP2 bind RIP1, and this interaction is important for NF-κB activation in the context of TNFR1 signaling [18, 19, 37–39]. We confirmed the binding of RIP1 to BIR3 (Fig 8) but not BIR2 (data not shown) of cIAP1 and cIAP2 by *in vitro* protein interaction experiments. We then compared the effects of selected SMAC analogues on this protein interaction. For these experiments, lysates of HEK293T cells expressing myc-RIP1 were incubated with GST-cIAP1-BIR3 or GST-cIAP2-BIR3 proteins in the presence of active cIAP1/cIAP2 inhibitory compounds **37** and **38** or the inactive negative control compound **40** that does not bind cIAP1 or cIAP2 (see Table 4; compounds **15** (**8q**), **16** (**2b**), and **23** (**8h**) from [22]; **37** from [29]; **38** from [40]; and **39** from [21]). The active compounds inhibited binding of myc-RIP1 to GST-cIAP1-BIR3 and GST-cIAP2-BIR3 protein, while the negative control did not (Fig 8). Thus, our SMAC mimetics that bind the BIR3 domains of cIAP1 and cIAP2 compete for RIP1 binding, which may contribute to their cytotoxicity.

**Fig 9:**
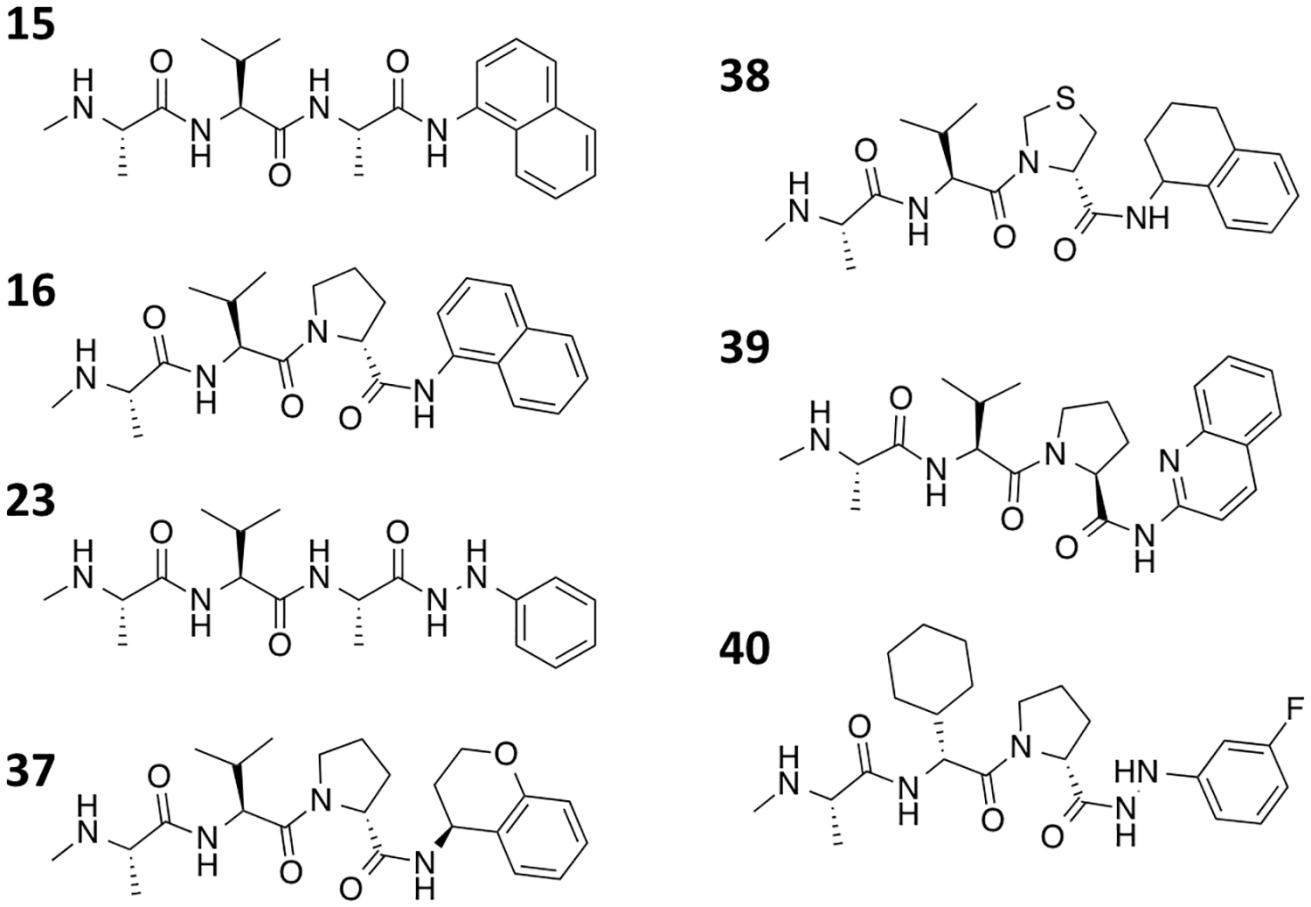
Structures of compounds described in Table 4.

## Discussion

The human genome contains 8 genes encoding IAP family proteins, altogether constituting 16 BIR domains, of which at least 9 bind SMAC [41]. Most efforts to generate apoptosis-promoting anticancer compounds based on mimicking SMAC have focused on XIAP as a target, due to both the technical ease of producing abundant quantities of recombinant SMAC peptide-binding BIR3 domain of XIAP, and the prominent functional role of XIAP in blocking caspase activation induced by TNF family members Fas (CD95) and TRAIL Receptors (CD261 and CD262). However, for optimizing the therapeutic index and combination therapeutic strategies, it may be important to define the spectrum of reactivity of compounds against different BIRs, particularly since different BIRs often play distinct roles in cellular processes [discussed in [42–46]].

Here, we have expanded the characterization of a series of SMAC mimicking compounds to determine their effects on cIAP1 and cIAP2 in addition to our previously published effects on XIAP [23]. XIAP is known for its ability to bind and inhibit effector caspases (caspases-3 and -7) *via* its BIR2 domain [47, 48] as well as the apical caspase in the mitochondrial pathway (caspase-9) via BIR3 [49], making it a particularly effective blocker of cell death induced by TNF receptor family members Fas (CD95), TRAIL-R1 (DR4; CD261), and TRAIL-R2 (DR5; CD262). In addition to binding caspases [50], cIAP1 and cIAP2 also interact with TRAF2, which mediates their recruitment to TNFR1 (CD120a) and TNFR2 (CD120b) [51]. The BIR3 domains of cIAP1 and cIAP2 bind the kinase RIP1, while their E3 ligase activity mediates ubiquitination of RIP1, stimulating NF-κB activation and suppressing activation of caspase-8 [19]. Induction of auto-ubiquitination and subsequent targeting for degradation of cIAP1 and cIAP2 by SMAC mimetics has been described previously [52, 53] and is further supported by our work. Thus, for purposes of sensitizing tumor cells to the cytotoxic actions of TNFR1 agonists such as TNF and LT-α, the proteins cIAP1 and cIAP2 are critical targets. In this regard, TNF and LT-α are commonly produced in the tumor microenvironment (reviewed in [54, 55]) and a modified version of recombinant TNF that preferentially localizes to tumors [56] is in Phase II/III clinical trials as a candidate cancer therapeutic [57].

Among our previously published XIAP inhibitory compounds [23], we identified SMAC analogues that also bind the BIR domains of cIAP1 and cIAP2. In fact, some compounds such as **1**, **2**, and **3** exhibited higher affinity binding to cIAP1 and cIAP2 than XIAP, as estimated by their relative K_i_ values. As predicted, the ability of our compounds to compete with SMAC peptide for binding to the BIR3 domains of cIAP1 and cIAP2 correlated closely with ability to sensitize tumor cells to cytotoxicity induced by TNF and LT-α. Although the mechanisms of cellular activity were not specifically evaluated here, we observed that exemplary active compounds (a) do not exhibit cytotoxicity in the absence of a complementary agent, such as TNF or LT-α; (b) cause reductions in cellular levels of cIAP1 protein; and (c) inhibit RIP1 binding to the BIR3 domains of cIAP1 and cIAP2. These characteristics are consistent with interference by active compounds of the participation of cIAP1 and cIAP2 in TNFR1 signaling. The contributions of our SMAC mimetics to RIP1 mediated effects can be ascribed to the direct inhibition of RIP1 binding to cIAP1-Bir3 or cIAP2-Bir3, as we observed in our assay, or the inhibitor mediated degradation and elimination of cIAP1 and cIAP2 as a RIP1 binding partner. Either mechanism allows the liberation of RIP1 to form the ripoptosome [58] and ultimately leads to necroptosis [58, 59]. Detailed further investigation might be able to clearly deconvolute the respective contributions.

In summary, we demonstrated that competitive displacement of SMAC from the BIR3 domains of cIAP1 and cIAP2 correlates closely with sensitization of tumor cells to TNFR1 agonists, TNF and LT-α. Optimization of SMAC mimicking compounds for targeting the BIR3 domains of cIAP1 and cIAP2 therefore represents a promising strategy for rendering selected types of malignant cells sensitive to cytokines such as TNF and LT-α. As such, rather than promoting tumor cell survival in the tumor microenvironment, TNF and LT-α may become cytotoxic in the presence of SMAC mimetics targeting cIAP1 and cIAP2. Our data support the findings of Beug *et al*. [60, 61], Etemadi *et al*. [62], and Benetatos *et al*. [63], who described the potential advantages and disadvantages of inciting a "cytokine storm" and generally concluded that there may be clinical benefit in exploring the combination of cytokines with Smac mimetics.

Finally, the assays described here may prove useful in aiding medicinal chemistry campaigns directed at optimizing compounds that bind cIAP1 and cIAP2 as cytokine sensitizers for tumor therapy.

## Supporting Information

S1 Prism Data of all graphs shown in Fig 5. (PZF)
